# Trichloroethylene-Induced Gene Expression and DNA Methylation Changes in B6C3F1 Mouse Liver

**DOI:** 10.1371/journal.pone.0116179

**Published:** 2014-12-30

**Authors:** Yan Jiang, Jiahong Chen, Jian Tong, Tao Chen

**Affiliations:** 1 Department of Physiology, School of Biology and Basic Medical Sciences, Soochow University, Suzhou, China; 2 Department of Toxicology, School of Public Health, Soochow University, Suzhou, China; 3 Jiangsu Key Laboratory of Preventive and Translational Medicine for Geriatric Diseases, Soochow University, Suzhou, China; Florida International University, United States of America

## Abstract

Trichloroethylene (TCE), widely used as an organic solvent in the industry, is a common contaminant in air, soil, and water. Chronic TCE exposure induced hepatocellular carcinoma in mice, and occupational exposure in humans was suggested to be associated with liver cancer. To understand the role of non-genotoxic mechanism(s) for TCE action, we examined the gene expression and DNA methylation changes in the liver of B6C3F1 mice orally administered with TCE (0, 100, 500 and 1000 mg/kg b.w. per day) for 5 days. After 5 days TCE treatment at a dose level of 1000 mg/kg b.w., a total of 431 differentially expressed genes were identified in mouse liver by microarray, of which 291 were up-regulated and 140 down-regulated. The expression changed genes were involved in key signal pathways including PPAR, proliferation, apoptosis and homologous recombination. Notably, the expression level of a number of vital genes involved in the regulation of DNA methylation, such as Utrf1, Tet2, DNMT1, DNMT3a and DNMT3b, were dysregulated. Although global DNA methylation change was not detected in the liver of mice exposed to TCE, the promoter regions of Cdkn1a and Ihh were found to be hypo- and hypermethylated respectively, which correlated negatively with their mRNA expression changes. Furthermore, the gene expression and DNA methylation changes induced by TCE were dose dependent. The overall data indicate that TCE exposure leads to aberrant DNA methylation changes, which might alter the expression of genes involved in the TCE-induced liver tumorgenesis.

## Introduction

Trichloroethylene (TCE) is widely used in the industry as an organic solvent for metal degreasing and in the production of chlorinated chemical compounds. It is a common contaminant in air, soil, water and a variety of foods [1]. Chronic TCE exposure was reported to induce hepatocellular carcinoma (HCC) and renal cell carcinoma in mice and rats, respectively [2]. Based on the strong association between occupational exposure and kidney cancer in humans, TCE was upgraded as human carcinogen (class I) in 2012 [3]. Liver is the main organ for TCE metabolism, and occupational exposure can cause liver damage [1]. A recent study also revealed a significant higher rate of liver cancer in TCE-exposed workers compared to normal people [4].

Till now, TCE’s carcinogenic mode of action (MOA) remain elusive [5]. A mutagenic MOA has been suggested for TCE induced kidney tumor, possibly due to the genotoxic metabolites of TCE formed via glutathione conjugation in kidney [6]. Biotransformation of TCE occurs in the liver mainly through cytochrome p450 enzymes, and tricarboxylic acid (TCA) is the major metabolite [1], [7]. Neither TCE nor TCA, however, has been confirmed to be mutagenic [1], therefore the role of non-genotoxic mechanisms in TCE hepatocarcinogenesis should be considered.

Non-genotoxic carcinogens have a wide variety of mechanisms of cancer induction, such as alteration of proliferation and apoptosis, peroxisome proliferation and epigenetic changes. Disturbance in the balance between proliferation and apoptosis is a hallmark of tumorgenesis, and irregular liver regeneration may be a common mechanism for hepatocarcinogenesis regardless of etiology [8], [9]. Hyperactivation of PPARα-induced peroxisome proliferation have also been suggested to contribute to the development of liver cancer in rodents [6]. At present, HCC is recognized as both a genetic and epigenetic disease. Increasing experimental data indicate that epigenetic regulation of gene expression by DNA methylation, histone modifications and microRNA plays fundamental roles at all stages of liver carcinogenesis [10].

DNA methylation is the most well researched epigenetic form. In vertebrates, DNA methylation occurs by the covalent addition of a methyl group to cytosine residues in CpG dinucleotides, which is catalyzed by DNA methyltransferases (DNMTs) with S-adenosylmethionine (SAM) as the main methyl donor. Global DNA hypomethylation and gene specific hypermethylation represent common early molecular events in most tumors [11]. In mouse liver, non-genotoxic carcinogens such as phenobarbital and choline-methionine decient diet could induce changes in DNA methylation resulting in alteration of gene expression [12]–[14]. So far, only Tao *et* *al.* reported hypomethylation of Jun and Myc promoter regions in the liver of mice treated with TCE [15].

The aim of this study is to understand the role of non-genotoxic mechanisms, especially DNA methylation, in TCE hepatocarcinogenesis. We first performed genome-wide gene expression analysis in the liver tissue of mice following oral administration with TCE to provide a clue for identification of diverse MOAs. The DNA methylation statuses of the promoter regions of a number of expression-changed genes as well as the global DNA methylation levels were then examined. We found that TCE induced dramatic changes in the expression of genes involved in key signal pathways including in the regulation of DNA methylation. Furthermore, TCE could cause both hypo- and hypermethylation on the promoter regions of single-copy genes which might contribute to the aberrant transcriptional changes.

## Materials and Methods

### Chemicals

TCE (CAS 79-01-6, ≥99.5% pure) and restriction enzymes were purchased from Adamas-beta (Shanghai, China) and Thermo Fisher Scientific (Waltham, MA, USA), respectively. The rest of chemicals were obtained from Sigma-Aldrich (St Louis, MO, USA) unless mentioned otherwise.

### Ethics Statement

All procedures involving animals were reviewed and approved by the Institutional Animal Care/User Ethical Committee of Soochow University, Suzhou City, China. (approval number A75–2013).

### Animal treatment

Adult B6C3F1 male mice, 6–7 weeks of age, were obtained from Model Animal Research Center of Nanjing University (Nanjing, China). They were kept in animal facility maintaining 22+/−1°C temperature, 50+/−5% relative humidity and 12-hour light-dark cycles. Commercial diet (Suzhou Shuangshi Laboratory Animal Feed Science Co. Ltd., Suzhou, China) and water were provided ad libitum. After 7 days quarantine period, twenty mice were distributed in 4 groups with 5 mice in each of them, and were dosed by gavage with TCE at doses of 0, 100, 500 and 1000 mg/kg b.w. in corn oil once a day for 5 days. No adverce effects was found during the treatment. Approximately 100 minutes after the last treatment, all mice were sacrificed by cervical dislocation. Form each mouse, liver was excised rapidly, frozen in liquid nitrogen, and stored at −80°C.

### Cell culture and 5-aza-2′-deoxycytidine (5-aza) treatment

BNL CL.2 mouse liver cells were obtained from the American Type Culture Collection (ATCC TIB-73, Manassas, VA, USA) and were maintained in Dulbecco’s minimum essential medium supplemented with 5% fetal bovine serum in a humidified atmosphere of 5% CO2 at 37°C. For treatment with 5-aza (A3656, Sigma), 210^5^ cells were seeded into each well of 6 well plates and treated with 5 µM 5-aza (dissolved in DMSO) or with DMSO as control for 72 h.

### DNA, RNA and protein extraction

Genomic DNA, RNA and protein were purified from liver tissue using TIANprep DNA-RNA-Protein Isolation kit (Tiangen, Beijing, China). Turbo DNA-free kit (Thermo Scientific) was used to remove genomic DNA contamination in purified RNA samples. The concentration and the quality of both DNA and RNA were assessed by ultraviolet (UV) absorbance using a NanoDrop ND-2000 spectrophotometer (Thermo Scientific). The integrity of RNA was determined using Agilent Bioanalyzer 2100 (Agilent Technologies, Santa Clara, CA, USA).

### Microarray analysis

The Agilent Mouse Gene Expression kit (8×60K, Design ID: 028005, Santa Clara, CA, USA) was used which included 39430 mRNAs and 16251 lincRNAs. The sample labeling, microarray hybridization and washings were performed as described in manufacturer’s instructions. Briefly, total RNAs were transcribed to double strand cDNA and then, synthesized into cRNA and labeled with Cyanine-3-CTP. The labeled cRNAs were hybridized onto the microarray. After washing, the arrays were scanned by the Agilent Scanner G2505C (Agilent Technologies). Feature Extraction software (version10.7.1.1, Agilent Technologies) was used to analyze microarray images to obtain the raw data and basic analysis was completed using Genesrping. For this, the raw data was normalized with the quantile algorithm. The probes with at least 100% of the values in any 1 out of all conditions were classified as “detected” and these were chosen for further analysis. Differentially expressed genes were then identified through fold change as well as P value obtained from the t-test. The threshold set for up- and down-regulated genes was a fold change of ≥2.0 and a P value ≤0.05. This was followed by gene ontology (GO) and Kyoto Encyclopedia of Genes and Genomes (KEGG) analyses to determine the role of differentially expressed mRNAs. Finally, Hierarchical Clustering was performed to display the distinguishable expression pattern of the genes among samples. The gene expression results have been deposited in the National Center for Biotechnology Information Gene Expression Omnibus (accession no.: GSE58819).

### Quantitative RT-PCR

The first stand cDNA was generated using SuperScript Reverse Transcriptase (Invitrogen, Carlsbad, USA). For quantitative PCR (qPCR), the standard two-step cycling and dissociation programs were performed using ABI Prism 7500 system (Applied Biosystems, Foster City, CA, USA). Primers were designed using oligo 5. Primer sequences and the corresponding annealing temperatures are listed in S1 Table. Expression level of differentially expressed genes was normalized to that of GAPDH and LDHA using the comparative threshold cycle (Ct) method (2^ΔΔCt^) as described previously [16].

### Western blot

The protein concentration was determined using a Bradford assay, and equal amounts of 15 µg of protein per sample were loaded and separated on a 10% SDS-PAGE gel. The proteins were then transferred to a PVDF membrane (Merck millionpore, Darmstadt, Germany). After blocking in 5% (w/v) BSA in TBS containing 0.1% (v/v) Tween-20 for 2 h, the membranes were probed with primary antibodies against Dnmt1(1∶1000, final concentration at 1.4 µg/ml) and Actin (1∶5000, final concentration at 0.2 µg/ml) overnight at 4°C. Both antibodies were purchased from Sangon biotech, Shanghai, China. The blots were developed with enhanced chemiluminescence regents. The protein bands were quantified using Gene Tools (Syngene, Cambridge, UK).

### Bisulfite sequencing and combined bisulfite restriction analysis (COBRA)

500 ng of genomic DNA from each mouse liver tissue was treated with bisulfite using EZ DNA Methylation-Gold Kit (Zymo Research, Orange, CA, USA) followed by PCR. The primers used were listed in S1 Table. For bisulfite sequencing, the PCR products of three control or TCE treated samples were pooled and cloned into TA-cloning vector (Tiangen), and individual clones were sequenced using an automatic sequencer (ABI 3730XL). For COBRA, the PCR products were digested with the corresponding restriction enzymes and analyzed on 2% agarose gel as described earlier [16].

### Liquid chromatography/tandem mass spectrometry (LC–MS/MS)

DNA hydrolysis and LC–MS/MS analyses were performed as described earlier [17]. Briefly, 5 µg genomic DNA from each mouse liver tissue was hydrolyzed by 5 U nuclease P1 for 1 h followed by treatment with 1 U alkaline phosphatase for 30 min at 37°C. Then, the DNA hydrolysate was diluted with double distilled water before loading to Agilent 1100 series LC coupled to an API 3000 triple quadrupole mass spectrometer equipped with a Turbo Ion Spray source (Applied Biosystems). A Thermo 150 mm ×210 mm, 5 µm column was used for separation with a mobile phase consisting of 0.1% formic acid and methanol. The quantity of 2-deoxycytosine (dC) and 5-methyldeoxycytidine (5-mC) were obtained using external standards. The content of 5-Me-dC was calculated as the ratio of (5-mC)/((5-mC)+(dC)).

### Statistical analyses

The data obtained in the liver tissue of TCE-exposed mice were compared to those in control mice. Data analysis was performed by one-way analysis of variance (ANOVA) followed by Dunnett’s multiple comparison test. For comparison of two groups, a student’s t-test was used. Statistical significance was defined as p<0.05. The results presented are mean +/− standard deviations (SD).

## Results

### Treatment of TCE at 1000 mg/kg b.w. induced dramatic gene expression changes in mouse liver

**Figure 1:**
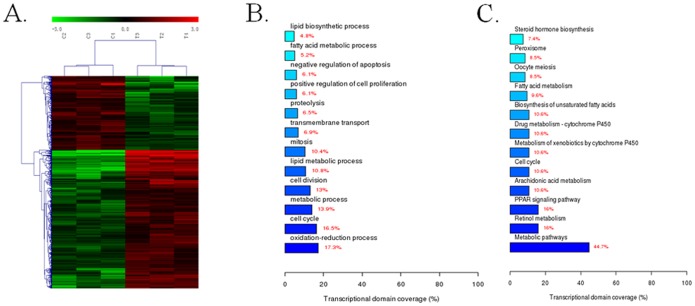
The effect of TCE on mouse liver transcriptome by microarray (n = 3). A) Heat map of mouse liver mRNAs affected by TCE at a dose level of 1000 mg/kg b.w. B) Go biological process annotations. C) Enriched pathways of the expression changed genes by KEGG.

Microarray data showed that the expression of 431 mRNAs were significantly changed (>2-fold expression change) after 5 days treatment of TCE at 1000 mg/kg b.w., of which 291 were up-regulated and 140 down-regulated (Fig. 1A and S2 Table). Biological functional annotation of the expression changed genes showed significant enrichment of clusters related to oxidation-reduction process (17.3%), cell cycle (16.5%), metabolic process (13.9%) and cell division (13%) (Fig. 1B). Notably, genes such as Jun, Ihh (Indian Hedgehog) and Cdkn1a were involved in both positive regulation of cell proliferation and negative regulation of apoptosis (Fig. 1B and S2 Table).

The KEGG analysis indicates that a large number of expression changed genes participate in metabolic pathways, especially in lipid and xenobiotics/drugs metabolism (Fig. 1C and S2 Table). Genes that target peroxisome proliferator-activated receptor (PPAR), proliferation and apoptosis signaling pathways were also significantly up-regulated (Fig. 1C and S2 Table). Furthermore, the mRNA expressions of Rad51b, Rad51 and Rad51ap1 were significantly increased, indicating disturbances in homologous recombination (HR) in the liver of mice exposed to TCE (S2 Table). For genes involved in the regulation of DNA methylation, only Uhrf1, which serves as a fidelity factor for the maintenance of the DNA methylation pattern, showed over two fold significant expression change (S2 Table). Three key genes responsible for DNA methylation or demethylation (Tet2, Dnmt3a and Dnmt3b) showed significant but less than 2 fold expression change (S2 Table).

**Figure 2:**
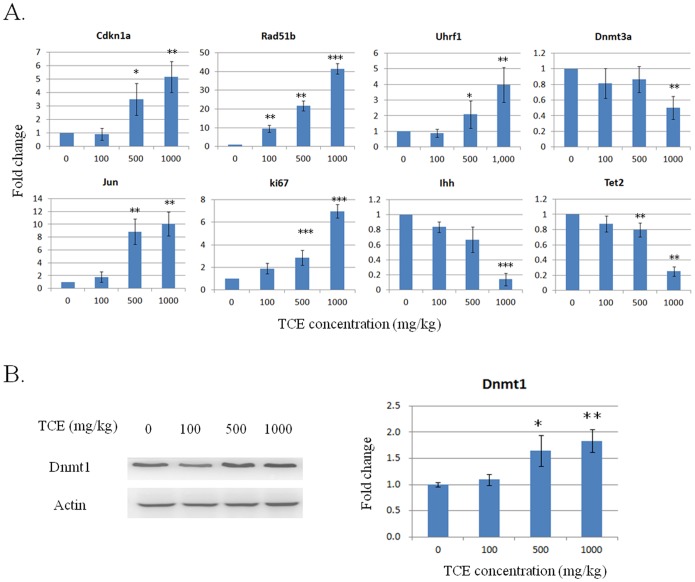
Selected gene expression changes in the liver of mice exposed to different doses of TCE (0, 100, 500 and 1000 mg/kg b.w.) (n = 3). A) qPCR analysis of selected mRNA expression changes in mouse liver. B) Western analysis of Dnmt1 protein expression levels. Fold expression at each dose was calculated against the nonexposed samples (TCE dose level at 0 mg/kg). *, p<0.05; **, p<0.01. ***, p<0.001.

Using qPCR, we validated seven out of eight of the genes with over 2 fold significant expression change, viz., Jun, Cdkn1a, Rad51b, Uhrf1, Mki67, Svil and Ihh (Table 1). The mRNA expression levels of DNA methylation modification genes Tet2, Dnmt3a and Dnmt3b showed significant but less than two fold down-regulation in our microarray results, while qPCR data showed over two times significant changes for all the three genes (Table 1). For Dnmt1 and Myc, no transcriptional expression change was detected by either microarray or qPCR (Table 1). However, the protein level of Dnmt1 was significantly increased in the liver of mice after TCE exposure (Fig. 2B).

**Table 1:** Comparison of mRNA expression changes detected by microarray and qPCR in mouse liver exposed to TCE at a dose level of 1000 mg/kg b.w. (n = 3).

mRNA	Microarray		qPCR	
	Fold change	p-value	Fold change	p-value
Jun	5.72	0.0044	10.08	0.0044
Cdkn1a	3.99	0.0109	5.16	0.0056
Rad51b	47.29	0.0002	41.36	0.0005
Uhrf1	5.04	0.0166	3.97	0.0065
Mki67	7.11	9.42E-05	6.97	0.0002
Svil	−3.45	0.0489	−3.03	0.0421
Cnn1	−5.26	0.0323	1.77	0.2293
Ihh	−4.17	0.0012	−7.06	0.0009
Tet2	−1.4	0.0171	−3.95	0.0093
Dnmt3a	−1.61	0.0022	−2.03	0.0052
Dnmt3b	−1.67	0.0241	−2.23	0.0201
Dnmt1	1.03	0.7328	1.90	0.1066
Myc	1.51	0.4495	1.02	0.2129

### The TCE-induced gene expression change was dose dependent

We examined the mRNA expression changes of eight genes in mouse liver treated with TCE at different dose levels and found a dose dependent trend (Fig. 2A). Especially, a significant mRNA expression increase of Rad51b was found in mouse liver treated with TCE at 100 mg/kg b.w. (Fig. 2A). The TCE-induced increase in the protein level of Dnmt1 was also dose dependent (Fig. 2B).

### TCE induced both hypo- and hypermethylation on the promoter regions of single-copy genes

**Figure 3:**
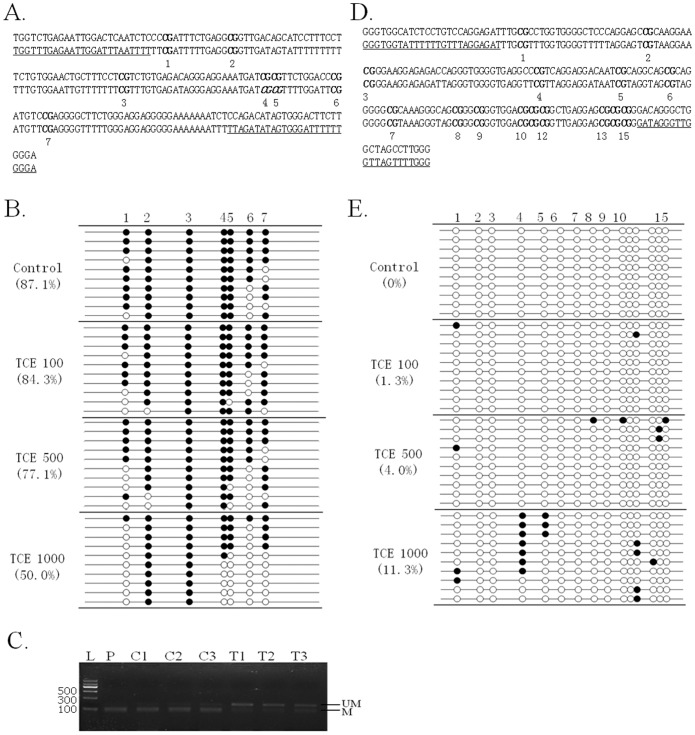
DNA methylation status of the promoter regions of Cdkn1a and Ihh in the liver of mice exposed to different doses of TCE (0, 100, 500 and 1000 mg/kg b.w.) (n = 3). A, D) Nucleotide sequences of Cdkn1a and Ihh promoter region fragments (upper strands) and the corresponding bisulphite-converted sequences (lower strands). CpG dinucleotides are numbered and marked in bold. The restriction enzyme cut sites are marked in italic. Primer sequences are underlined. B, E) Bisulfite sequencing of the Cdkn1a and Ihh promoter regions. Open and closed circles indicate unmethylated and methylated CpG sites respectively. Percent methylation is shown in parentheses. C) COBRA result of the promoter region of Cdkn1a at CpG4-5(CGCG) by *BstUI* 184 bp (84/100); L: Tiangen DNA ladder II; P, positive control by treating mouse genomic DNA with M.SssI. C, liver samples from mice exposed to corn oil; T, liver samples from mice exposed to TCE at 1000 mg/kg b.w. M, methylation; UM, unmethylation.

**Figure 4:**
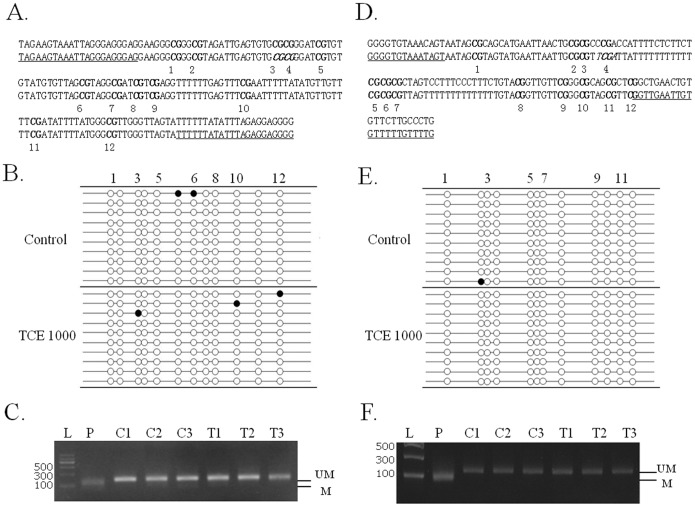
DNA methylation status of the promoter regions of Jun and Myc in the liver from mice exposed to TCE at 0 or 1000 mg/kg b.w. (n = 3). A, D) Nucleotide sequences of Jun and Myc promoter region fragments (upper strands) and the corresponding bisulphite-converted sequences (lower strands). CpG dinucleotides are numbered and marked in bold. The restriction enzyme cut sites are marked in italic. Primer sequences are underlined. (B, E) Bisulfite sequencing of the Jun and Myc promoter regions. Open and closed circles indicate unmethylated and methylated CpG sites respectively. C) COBRA result of the promoter region of Jun at CpG3,4 (CGCG) by *BstUI* 171 bp (49/122). F) COBRA result of the promoter region of Myc at CpG4 (TCGA) by *TaqI* 132 bp (88/43). L: Tiangen DNA ladder; P, positive control by treating mouse genomic DNA with M.SssI. C, control liver samples; T, liver samples treated with TCE at 1000 mg/kg b.w. M methylation; UM, unmethylation.

To examine if the gene expression changes induced by TCE were due to DNA methylation alteration, we examined the DNA methylation status of the promoter regions of Jun, Myc, Ihh and Cdkn1a. As shown in Fig. 3A, B, the DNA methylation level of the promoter region of Cdkn1a was dose dependently decreased in TCE treated mouse liver. For samples treated with TCE at a dose level of 1000 mg/kg b.w., the reduction of DNA methylation at CpG sites 4 and 5, which locate in a *BstUI* recognizition site, was confirmed by COBRA (Fig. 3C). On the other hand, the DNA methylation level of the promoter region of Ihh was dose dependently increased (Fig. 3D, E). No DNA methylation change in the promoter regions of Jun and Myc was detected in mouse liver exposed to TCE at 1000 mg/kg b.w. (Fig. 4). Furthermore, the DNA demethylating agent 5-aza increased the mRNA expression level of both Cdkn1a and Jun in BNL CL.2 mouse hepatocytes (Table 2).

**Table 2:** mRNA expression changes in BNL CL.2 mouse liver cells after 5aza treatment (n = 3).

mRNA	Fold change	p-value
Cdkn1a	2.10	0.0312
Jun	3.70	0.0368
Myc	1.16	0.2129
Ihh	1.96	0.0769
Rad51b	−1.65	0.2620

### TCE treatment did not affect global DNA methylation

**Figure 5:**
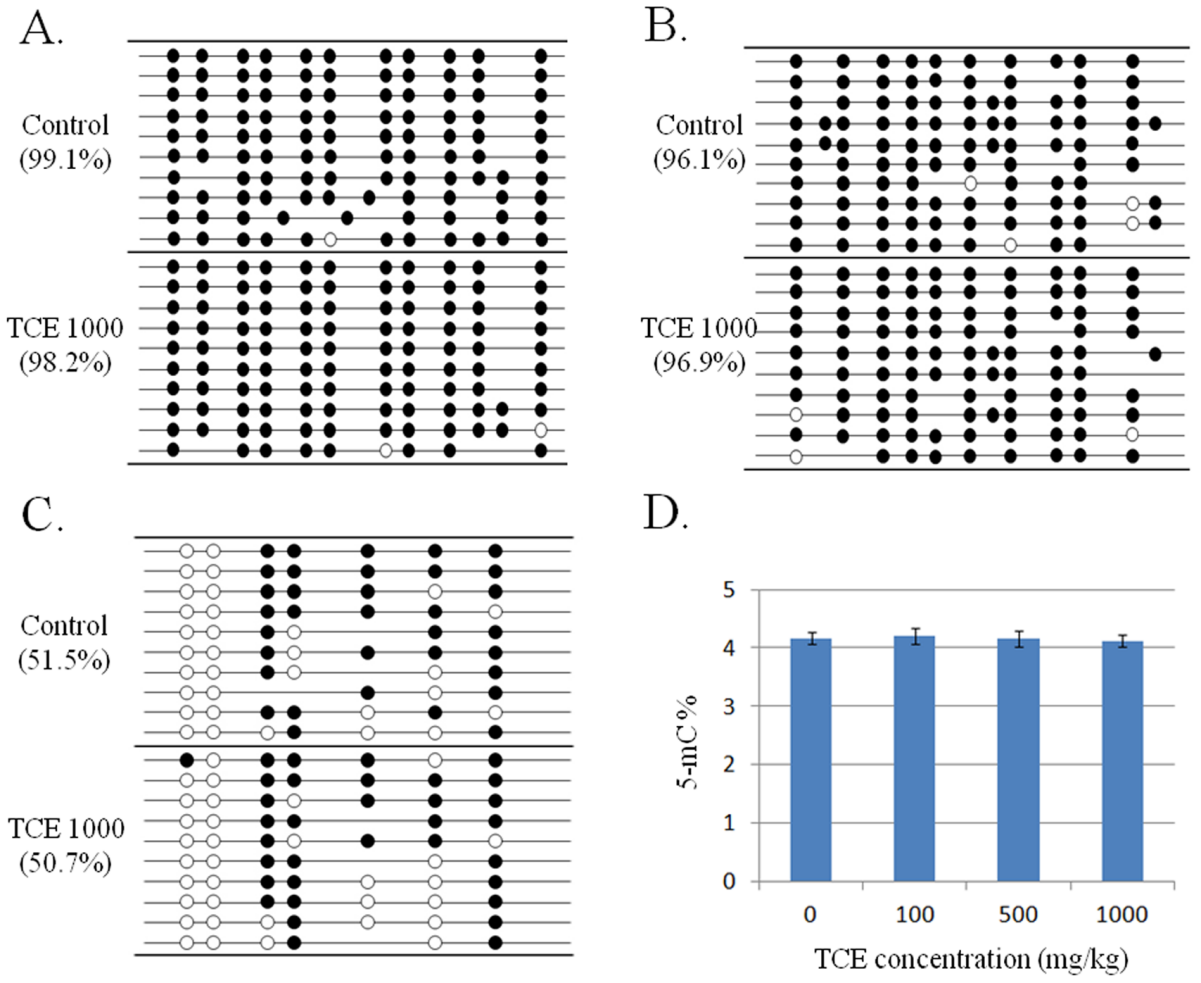
Global DNA methylation status of the liver of mice exposed to TCE. A–C) Bisulfite sequencing of the LINE-1, LAP-LTR and SINE B1 repetitive elements in the liver of mice treated with TCE at 0 or 1000 mg/kg b.w. Open and closed circles indicate unmethylated and methylated CpG sites respectively. Some sites are absent from the sequences in some clones due to mutations in the particular copies of the repetitive sequences. Percent methylation is shown in parentheses. D) The content of 5-mC detected by LC-MS/MS. (n = 5).

We next detected the DNA methylation status of three major repetitive DNA elements, including LINE-1, LAP-LTR (intracisternal A particle long terminal repeat retrotransposons) and SINE B1, which could be served as indicators for global DNA methylation change [18]. As shown in Fig. 5A–C and S1 Fig., none of the repetitive elements showed DNA methylation change in mouse liver exposed to TCE at 1000 mg/kg b.w. Because the bisulfite treatment technique we used to detect gene specific methylation cannot distinguish the difference between 5-hydroxymethylcytosine (5-hmC) and 5-methylcytosine (5-mC), we further performed LC-MS/MS to directly detect global 5-mC level and found no significant change in mouse liver after TCE exposure (Fig. 5D).

## Discussion

TCE has been reported as a hepatic carcinogen in experimental animals as well as in human epidemiologic studies [1], [2], [4], but the exact molecular mechanism(s) involved in TCE-induced hepatocarcinogenesis remain elucidated. In this study, we observed the dysregulation of a number of genes involved in PPAR, proliferation and apoptosis pathways in mouse liver after 1000 mg/kg TCE exposure. Especially, the expression of Mki67, a cell proliferation-associated nuclear marker, increased dose dependently in TCE treated mouse liver. TCA, the main liver metabolite of TCE, is a ligand for peroxisome proliferator activated receptors (PPARs), and peroxisome proliferation has been reported in mouse liver after TCE exposure [7], [19]. Hepatocyte hyperplasia and increased liver-to-body weight have also been frequently observed in TCE-exposed mouse liver [7], [20].

It is interesting that the mRNA expression levels of three HR-related genes (Rad51, Rad51b and Rad51ap1) were dramatically increased in the liver of mice administered with TCE at 1000 mg/kg b.w. For Rad51b, TCE exposure at a dose level of 100 mg/kg b.w. could lead to a significant mRNA overexpression. Moreover, the TCE-induced mRNA overexpression of Rad51b was dose dependent. HR catalyzed by the Rad51 is a major mechanism involved in the elimination of DNA double-strand breaks, and dysfunction in HR results in inappropriate recombination and genomic instability. Rad51b promotes the assembly of Rad51 nucleoprotein filaments while Rad51ap1interacts with Rad51 to enhance its recombinase activity [21]–[23]. To our knowledge, the current study demonstrates for the first time that TCE exposure could dysregulate the expression of HR-related genes in mouse liver, which might have led to hyperactivity of HR resulting in genomic fragility and tumorgenesis.

The mRNA expression level of Cdkn1a and Ihh could be affected by the DNA methylation status of their promoter regions [24], [25]. Cdkn1a plays a key role in the p53-mediated cell cycle arrest. Over-expression of Cdkn1a has been observed in HCC tissues, which might promote liver regeneration and prevents pre-cancerous lesions from undergoing apoptosis, thereby contributing to oncogenesis [26], [27]. Ihh, a ligand in fetal morphogenic signaling pathway, has also been reported to be down-regulated in serious cancers [28]. Here, we demonstrated that the promoter regions of Cdkn1a and Ihh were hypo- and hypermethylated respectively in mouse liver after TCE exposure, which correlated negatively with their mRNA expression changes. The correlation between DNA hypomethylation and mRNA overexpression of Cdkn1a was confirmed by treating mouse hypatocytes with DNA demethylation agent 5-aza.

Tao *et* *al.* reported that TCE could induce DNA hypomethylation and mRNA overexpression of Jun and Myc in mouse liver [15]. Jun and Myc, two well-known oncogenes, are critical promoters of cellular proliferation. Dysregulated expression and activation of the two genes are frequently observed in cancers [29], [30]. In this study, the mRNA expression level of Jun was increased, but no DNA methylation change was found in its promoter region. Because bisulfite sequencing can only detect the DNA methlation status of a short DNA fragment, we cannot role out the possibility that TCE might cause DNA methylation change in other part of the Jun promoter. In fact, the mRNA expression level of Jun was increased in mouse liver cells after 5-aza treatment, indicating that the DNA methylation status of Jun can modulate its mRNA expression level in mouse liver. For Myc, we did not find any change in DNA methylation or mRNA expression. The inconsistence between our study and Tao *et* *al.* might be due to animal difference. Overexpression of Myc mRNA and protein has been reported in some mice strains but not in B6C3F1 mice [31].

It has been suggested that TCE induced DNA hypomethylation by depleting the availability of SAM, the main substrate for methylation reactions [32]. However, we found that TCE not only induced DNA hypomethylation of Cdkn1a, but also caused hypermethylation of Ihh in mouse liver after TCE exposure. In addition, the DNA methylation status of repetitive sequences as well as global 5-mC level was not changed. TCE induced hypermethylation of Serca2 promoter region has also been reported in cardiac myoblast cells and rat embryonic heart [33]. Thus, the TCE induced DNA methylation change cannot be simply explained by the shortage of SAM. We noticed that a number of genes involved in the regulation of DNA methylation (Uhrf1, Tet2, Dnmt1, Dnmt3a and Dnmt3b) showed significant expression changes in mouse liver after TCE exposure. Uhrf1, which has been reported to be over-expressed in HCC, can recruit the maintenance DNA methyltransferase Dnmt1 to repress tumor suppressor gene expression [34]. Tet2, a 5-methylcytosine oxidase, is involved in active DNA demethylation [35]. The overexpression of Uhrf1 and Dnmt1 as well as the down-regulation of Tet2 might account for the hypermethylation of the promoter region of Ihh and its mRNA down-regulation. On the other hand, the down-regulation of Dnmt3a and Dnmt3b, two *de novo* DNA methyltransferase, might be responsible for the hypomethylation of the promoter region of Cdkn1a and its mRNA overexpression. Further work need to be down to validate the contribution of aberrant expression of DNA methylation regulating genes to TCE-induced promoter DNA methylation changes.

In conclusion, our findings indicate that in mouse liver TCE-induced DNA methylation changes might disturb the transcriptional regulation of genes targeting various signal pathways that affect tumorgenesis. The aberrant expression of key genes involved in the regulation of DNA methylation could account for the TCE-induced DNA methylation alteration. Our research may shed light on non-genotoxic mechanism(s) in TCE-induced hepatocarcinogenesis, which is vital for the assessment of human relevance and existence of threshold.

## Supporting Information

S1 Fig **DNA methylation status of IAP promoter region by COBRA.** A) Nucleotide sequences of IAP promoter region fragments (upper strands) and the corresponding bisulphite-converted sequences (lower strands). CpG dinucleotides are numbered and marked in bold. The restriction enzyme *BstUI* cut sites are marked in italic. Primer sequences are underlined. B) COBRA result of the promoter region of IAP at CpG2,6 (TCAG) by 263 bp (41/54/168); L: Tiangen DNA ladderII; P, positive control by treating mouse genomic DNA with *M.SssI.* C, liver samples from mice exposed to corn oil; T, liver samples from mice exposed to TCE at 1000 mg/kg b.w. M, methylation; UM, unmethylation.
(DOC)

S1 Table **Primers used and the corresponding annealing temperatures (AT).**
(DOC)

S2 Table **mRNAs with over two fold significant expression change by microarray in mouse liver exposed to TCE at 1000 mg/kg b.w.**
(DOC)
