# Supplementary material for: Trichloroethylene-Induced Gene Expression and DNA Methylation Changes in B6C3F1 Mouse Liver
Source: PLoS One. 2014 Dec 30;9(12):e116179. doi: 10.1371/journal.pone.0116179 (PMC4280179; doi:10.1371/journal.pone.0116179)
Supplement: S1 Fig — DNA methylation status of IAP promoter region by COBRA. A) Nucleotide sequences of IAP promoter region fragments (upper strands) and the corresponding bisulphite-converted sequences (lower strands). CpG dinucleotides are numbered and marked in bold. The restriction enzyme BstUI cut sites are marked in italic. Primer sequences are underlined. B) COBRA result of the promoter region of IAP at CpG2,6 (TCAG) by 263 bp (41/54/168); L: Tiangen DNA ladderII; P, positive control by treating mouse genomic DNA with M.SssI. C, liver samples from mice exposed to corn oil; T, liver samples from mice exposed to TCE at 1000 mg/kg b.w. M, methylation; UM, unmethylation. (DOC) [file pone.0116179.s001.doc]

**Supplemental Figure 1. DNA methylation status of IAP promoter region by COBRA.**

A) Nucleotide sequences of IAP promoter region fragments (upper strands) and the corresponding bisulphite-converted sequences (lower strands). CpG dinucleotides are numbered and marked in bold. The restriction enzyme *BstUI* cut sites are marked in italic. Primer sequences are underlined. B) COBRA result of the promoter region of IAP at CpG2,6 (TCAG) by *TaqI* 263bp (41/54/168); L: Tiangen DNA ladder Ⅱ; P, positive control by treating mouse genomic DNA with *M.SssI.* C, liver samples from mice exposed to corn oil; T, liver samples from mice exposed to TCE at 1000mg/kg b.w.. M, methylation; UM, unmethylation.

**
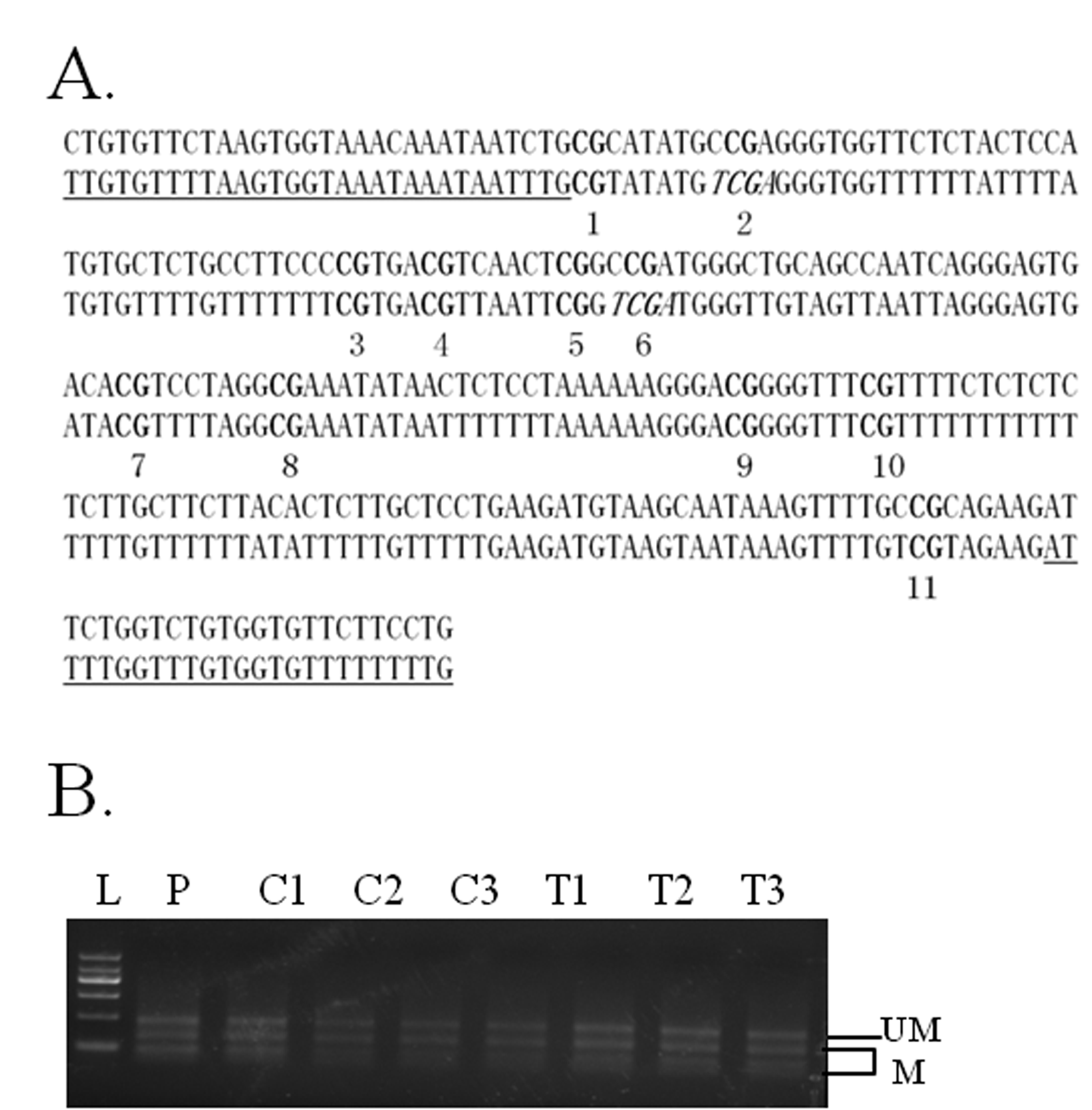
**
