# Supplementary material for: Trichloroethylene-Induced Gene Expression and DNA Methylation Changes in B6C3F1 Mouse Liver
Source: PLoS One. 2014 Dec 30;9(12):e116179. doi: 10.1371/journal.pone.0116179 (PMC4280179; doi:10.1371/journal.pone.0116179)
Supplement: S1 Table — Primers used and the corresponding annealing temperatures (AT). (DOC) [file pone.0116179.s002.doc]

**Supplemental Table 1.** **Primers used and the corresponding annealing temperatures (AT).**

|  | Gene Name | Sense | Antisense | PCR Length (bp) | AT (℃) |
| --- | --- | --- | --- | --- | --- |
| RT-PCR | Jun | AGCAGGGACCCATGGAAGTT | ATGCACAAGCAAAGGTCATCTTT | 92 | 60 |
|  | Cdkn1a | GACAGTGAGCAGTTGCG | CTCAGACACCAGAGTGC | 288 | 60 |
|  | Rad51b | GGGCTGTGGTCTACATCGAC | CTCGGCAAAGATGAACTCTA | 138 | 60 |
|  | Uhrf1 | CCACACCGTGAACTCTCTGTC | GGCGCACATCATAATCGAAGA | 155 | 60 |
|  | Myc | TCTCCACTCACCAGCACAACTACG | ATCTGCTTCAGGACCCT | 103 | 60 |
|  | Svil | GCTAGTGACTCATCGGCTGC | GGAGCCCGAACTATGGA | 178 | 60 |
|  | Ihh | CTCTTGCCTACAAGCAGTTCA | CCGTGTTCTCCTCGTCCTT | 156 | 60 |
|  | Tet2 | AGAGAAGACAATCGAGAAGTCGG | CCTTCCGTACTCCCAAACTCAT | 104 | 60 |
|  | Dnmt1 | AAGAATGGTGTTGTCTACCGAC | CATCCAGGTTGCTCCCCTTG | 178 | 60 |
|  | Dnmt3a | GAGGGAACTGAGACCCCAC | CTGGAAGGTGAGTCTTGGCA | 216 | 60 |
|  | Dnmt3b | AGCGGGTATGAGGAGTGCAT | GGGAGCATCCTTCGTGTCTG | 73 | 60 |
|  | Gapdh | AGGTCGGTGTGAACGGATTTG | TGTAGACCATGTAGTTGAGGTCA | 123 | 60 |
| BSP | Cdkn1a | TGGTTTGAGAATTGGATTTAATTTT | TCCCAAAAAATCCCACTATATCTAA | 184 | 55 |
|  | Ihh | GGGTGGTATTTTTTGTTTAGGAGAT | CCCAAAACTAACCAACCCTATC | 192 | 55 |
|  | Jun | TAGAAGTAAATTAGGGAGGGAG | CCCCTCCTCTAAATATAAAAAA | 171 | 55 |
|  | Myc | GGGGTGTAAATAGTAATAGT | CAAAACAAAAACACAATTCAACC | 132 | 55 |
|  | LINE-1- | GGGGTGTAAATAGTAATAGT | CAAAACAAAAACACAATTCAACC | 266 | 55 |
|  | IAP-LTR | TTGTGTTTTAAGTGGTAAATAAATAATTTG | CAAAAAAAACACACAAACCAAAAT | 263 | 55 |
|  | SINE B1 | AGTYGGGYGTGGTGG | CTTATAAACCAAACTAACCTC | 92 | 55 |
