# Supplementary material for: Trichloroethylene-Induced Gene Expression and DNA Methylation Changes in B6C3F1 Mouse Liver
Source: PLoS One. 2014 Dec 30;9(12):e116179. doi: 10.1371/journal.pone.0116179 (PMC4280179; doi:10.1371/journal.pone.0116179)
Supplement: S2 Table — mRNAs with over two fold significant expression change by microarray in mouse liver exposed to TCE at 1000 mg/kg b.w. (DOC) [file pone.0116179.s003.doc]

**Supplemental Table 2.** **mRNAs with over two fold significant expression change by microarray in mouse liver exposed to TCE at 1000 mg/kg b.w.. (n=3).**

| **ProbeName** | **p value** | **Fold change** | **Regulation** | **GeneSymbol** | **Genbank No.** |
| --- | --- | --- | --- | --- | --- |
| A_55_P2246900 | 0.00216 | 2.45 | down | A430017K17 | AK079696 |
| A_55_P2325038 | 0.00525 | 3.06 | down | Acsm2 | NM_001177977 |
| A_55_P2046550 | 0.01589 | 2.02 | down | Adamts8 | NM_013906 |
| A_55_P2153620 | 0.01098 | 5.69 | down | Ahnak | NM_001039959 |
| A_52_P16752 | 0.00581 | 2.22 | down | Aox3 | NM_023617 |
| A_52_P239086 | 0.00025 | 10.66 | down | Apol10a | NM_177744 |
| A_55_P2170881 | 0.04684 | 2.96 | down | Arhgap26 | NM_175164 |
| A_55_P2221236 | 0.00066 | 2.62 | down | AU024180 | AK085976 |
| A_55_P2339601 | 0.00669 | 2.17 | down | AW060742 | CO043442 |
| A_55_P2029106 | 0.04610 | 2.53 | down | Bmf | NM_138313 |
| A_55_P1969471 | 0.00579 | 2.25 | down | C2cd4d | NM_001136117 |
| A_55_P1954116 | 0.00276 | 2.08 | down | C8b | NM_133882 |
| A_51_P255295 | 0.00943 | 2.01 | down | C9 | NM_013485 |
| A_66_P110769 | 0.00100 | 3.23 | down | Cabyr | NM_027687 |
| A_55_P2117105 | 0.00771 | 3.14 | down | Ccnj | NM_172839 |
| A_51_P291501 | 0.01453 | 2.70 | down | Ccno | NM_001081062 |
| A_55_P1959122 | 0.00031 | 4.60 | down | Cd164l2 |  |
| A_51_P199135 | 0.00883 | 2.00 | down | Cd83 | NM_009856 |
| A_55_P2293013 | 0.00244 | 2.13 | down | Ces2a | NM_133960 |
| A_55_P2005213 | 0.01321 | 2.21 | down | Ces2c | NM_145603 |
| A_51_P478881 | 0.02770 | 2.93 | down | Ces4a | NM_146213 |
| A_52_P320193 | 0.00179 | 6.26 | down | Clec2h | NM_053165 |
| A_55_P2363030 | 0.03986 | 2.01 | down | Cmtm4 | NM_153582 |
| A_51_P350817 | 0.03229 | 5.14 | down | Cnn1 | NM_009922 |
| A_51_P501844 | 0.02627 | 8.25 | down | Cyp26b1 | NM_175475 |
| A_51_P498882 | 0.00064 | 2.04 | down | Cyp2c37 | NM_010001 |
| A_52_P154580 | 0.04108 | 2.19 | down | Cyp2c54 | NM_206537 |
| A_55_P1962661 | 0.00482 | 2.10 | down | Cyp2c67 | NM_001024719 |
| A_55_P2058433 | 0.00446 | 2.17 | down | Cyp2c68 | NM_001039555 |
| A_66_P127161 | 0.00820 | 2.15 | down | Cyp2u1 | NM_027816 |
| A_52_P302345 | 0.00042 | 2.14 | down | Cyp4v3 | NM_133969 |
| A_52_P84027 | 0.03404 | 5.65 | down | Cyp7a1 | NM_007824 |
| A_51_P461429 | 0.00667 | 2.69 | down | Cyp7b1 | NM_007825 |
| A_51_P252859 | 0.01786 | 2.35 | down | Cyr61 | NM_010516 |
| A_52_P204331 | 0.01567 | 2.62 | down | D630039A03Rik | NM_178727 |
| A_55_P2177899 | 0.00803 | 2.82 | down | Dact1 | NM_001190466 |
| A_55_P2003053 | 0.02215 | 2.20 | down | Dct | NM_010024 |
| A_52_P63905 | 0.00196 | 2.49 | down | Ddc | NM_016672 |
| A_51_P491667 | 0.00525 | 5.56 | down | Derl3 | NM_024440 |
| A_55_P1997211 | 0.04827 | 2.03 | down | Diras1 | NM_145217 |
| A_55_P2000148 | 0.00932 | 2.26 | down | Dnaic1 | NM_175138 |
| A_55_P2085955 | 0.00711 | 2.57 | down | Dnajb11 | NM_026400 |
| A_55_P2028243 | 0.00512 | 2.18 | down | Dnajc19 | NM_001026211 |
| A_51_P202050 | 0.02658 | 2.14 | down | Dtx1 | NM_008052 |
| A_55_P1956863 | 0.00061 | 2.30 | down | Egfr | NM_007912 |
| A_52_P588483 | 0.01075 | 2.27 | down | Fbln1 | NM_010180 |
| A_52_P423364 | 0.02223 | 2.61 | down | Fbxl7 | NM_176959 |
| A_55_P2095181 | 0.03010 | 2.99 | down | Fdxacb1 | AK135025 |
| A_55_P2040090 | 0.00015 | 2.11 | down | Fn3k | NM_001038699 |
| A_55_P2067533 | 0.00609 | 2.21 | down | Foxa2 | NM_010446 |
| A_55_P2035804 | 0.00018 | 2.56 | down | Fzd8 | NM_008058 |
| A_55_P1978825 | 0.00189 | 3.40 | down | Gm10556 | NR_045881 |
| A_55_P2024679 | 0.01029 | 2.46 | down | Gm10765 | XR_140521 |
| A_55_P2185330 | 0.02044 | 2.07 | down | Gm10914 | XR_141300 |
| A_66_P105032 | 0.00552 | 2.08 | down | Gm13889 | NM_001145034 |
| A_55_P2141058 | 0.01139 | 6.26 | down | Gm4477 | NM_001253910 |
| A_66_P140856 | 0.00115 | 4.59 | down | Gm4588 | XR_168429 |
| A_55_P1986341 | 0.00001 | 4.64 | down | Gm4956 | NR_002858 |
| A_55_P2143934 | 0.04847 | 2.15 | down | Gm9785 | BC147682 |
| A_55_P2186302 | 0.03856 | 2.01 | down | Gm9866 | NR_045870 |
| A_66_P106611 | 0.02869 | 2.82 | down | Gna14 | NM_008137 |
| A_52_P566840 | 0.00366 | 2.90 | down | Gpr110 | NM_133776 |
| A_55_P2263053 | 0.01721 | 2.33 | down | Hal | CK128530 |
| A_51_P465582 | 0.00226 | 2.16 | down | Hdhd3 | NM_024257 |
| A_55_P1969481 | 0.03044 | 4.10 | down | Hivep3 | NM_010657 |
| A_55_P2044369 | 0.02223 | 2.07 | down | Hs2st1 | NM_011828 |
| A_55_P1969665 | 0.02968 | 3.01 | down | Hsd3b4 | NM_001111336 |
| A_55_P2111980 | 0.00703 | 3.55 | down | Hsd3b5 | NM_008295 |
| A_51_P326043 | 0.00030 | 5.54 | down | Hydin | NM_172916 |
| A_51_P277088 | 0.00150 | 2.19 | down | Igfals | NM_008340 |
| A_55_P2152225 | 0.00123 | 4.19 | down | Ihh | NM_010544 |
| A_55_P2034033 | 0.00215 | 2.35 | down | Il12rb1 | NM_008353 |
| A_51_P346668 | 0.00671 | 2.44 | down | Irf5 | NM_012057 |
| A_52_P1092823 | 0.01848 | 2.74 | down | Irx1 | NM_010573 |
| A_55_P2145617 | 0.03575 | 2.62 | down | Krt80 | NM_028770 |
| A_55_P2064333 | 0.02436 | 5.66 | down | Lama3 | NM_010680 |
| A_55_P2126192 | 0.00327 | 2.17 | down | Lgr5 | NM_010195 |
| A_55_P2159264 | 0.00247 | 2.26 | down | Lifr | NM_001113386 |
| A_55_P2207342 | 0.01211 | 2.07 | down | LOC101056197 | XM_003945397 |
| A_52_P669005 | 0.01246 | 2.54 | down | Lrat | NM_023624 |
| A_51_P326685 | 0.03908 | 3.79 | down | Lrtm1 | NM_176920 |
| A_51_P265495 | 0.00997 | 2.39 | down | Ly6a | NM_010738 |
| A_55_P2058962 | 0.01206 | 2.14 | down | Mcm10 | NM_027290 |
| A_51_P455997 | 0.02690 | 4.63 | down | Meg3 | NR_027652 |
| A_66_P109220 | 0.00877 | 2.15 | down | Mettl20 | AK077292 |
| A_55_P2025343 | 0.00009 | 2.45 | down | Mup21 | NM_001009550 |
| A_51_P355943 | 0.00139 | 3.00 | down | Mvd | NM_138656 |
| A_55_P2058861 | 0.01476 | 2.15 | down | Mvk | NM_023556 |
| A_55_P2140453 | 0.00782 | 6.06 | down | Myh6 | NM_001164171 |
| A_55_P2071059 | 0.01970 | 2.14 | down | Nab2 | NM_008668 |
| A_51_P479230 | 0.00555 | 3.01 | down | Nat8 | NM_023455 |
| A_51_P515965 | 0.02239 | 2.59 | down | Nfe2 | NM_008685 |
| A_51_P234113 | 0.02190 | 2.85 | down | Nod1 | NM_172729 |
| A_55_P1979973 | 0.02838 | 2.25 | down | Nuak2 | NM_001195025 |
| A_51_P193336 | 0.01363 | 2.27 | down | Nucb2 | NM_001130479 |
| A_55_P2135153 | 0.00636 | 2.71 | down | Obp2a | NM_153558 |
| A_51_P391897 | 0.01971 | 2.21 | down | Olfr125 | NM_146290 |
| A_55_P2087876 | 0.04042 | 2.13 | down | Patz1 | AK166189 |
| A_55_P2075127 | 0.01701 | 2.07 | down | Pax2 | NM_011037 |
| A_66_P130887 | 0.01177 | 2.96 | down | Pcdh18 | NM_130448 |
| A_51_P397673 | 0.02517 | 2.05 | down | Pcsk9 | NM_153565 |
| A_52_P423247 | 0.03642 | 2.13 | down | Pde4b | NM_019840 |
| A_51_P195958 | 0.04138 | 2.43 | down | Phlda1 | NM_009344 |
| A_55_P2026275 | 0.03608 | 2.33 | down | Ppp1r1b | NM_144828 |
| A_52_P306357 | 0.00114 | 4.07 | down | Prok1 | BC042707 |
| A_55_P2100928 | 0.03913 | 2.05 | down | Ptgds | NM_008963 |
| A_51_P311904 | 0.00020 | 3.38 | down | Ptk2b | NM_172498 |
| A_55_P2094626 | 0.02483 | 2.51 | down | Ralgps1 | NM_175211 |
| A_55_P2027117 | 0.01583 | 2.12 | down | Rgs3 | NM_019492 |
| A_66_P125741 | 0.02984 | 3.37 | down | Ripply2 | NM_001037907 |
| A_55_P2009217 | 0.04632 | 2.27 | down | Rnu3b1 | NR_004415 |
| A_51_P435922 | 0.00547 | 3.15 | down | Rsph9 | NM_029338 |
| A_52_P532227 | 0.00579 | 2.07 | down | S1pr1 | NM_007901 |
| A_51_P209372 | 0.00194 | 2.66 | down | Sc4mol | NM_025436 |
| A_51_P280446 | 0.00012 | 2.25 | down | Sdf2l1 | NM_022324 |
| A_51_P387400 | 0.00272 | 10.82 | down | Serpina12 | NM_026535 |
| A_55_P1997003 | 0.01136 | 7.70 | down | Serpina4-ps1 | BC031891 |
| A_55_P2092219 | 0.00313 | 3.33 | down | Serpina9 | NM_027997 |
| A_51_P268094 | 0.01433 | 2.04 | down | Serpine2 | NM_009255 |
| A_55_P1994550 | 0.01092 | 2.17 | down | Shank1 | NM_001034115 |
| A_55_P2141008 | 0.00328 | 2.04 | down | Siglech | AK080658 |
| A_55_P2143923 | 0.00312 | 2.24 | down | Slc13a2 | NM_022411 |
| A_55_P2067518 | 0.00090 | 2.48 | down | Slc13a3 | NM_054055 |
| A_55_P1955412 | 0.00040 | 3.13 | down | Slc1a2 | NM_011393 |
| A_55_P2139087 | 0.00053 | 2.28 | down | Slc22a28 | NM_001013820 |
| A_55_P2169124 | 0.03104 | 2.33 | down | Slc22a30 | AK040781 |
| A_55_P2207186 | 0.03981 | 2.20 | down | Slc30a7 | NM_023214 |
| A_51_P335569 | 0.00348 | 2.37 | down | Slco1a4 | NM_030687 |
| A_55_P1989673 | 0.00189 | 2.35 | down | Slco2a1 | NM_033314 |
| A_66_P113892 | 0.02776 | 2.07 | down | Smim6 | NM_001162998 |
| A_55_P1975185 | 0.00279 | 2.60 | down | Sqle | NM_009270 |
| A_51_P471458 | 0.02060 | 2.17 | down | Sult5a1 | NM_020564 |
| A_52_P42194 | 0.04894 | 3.48 | down | Svil | AK041925 |
| A_55_P1994733 | 0.01786 | 2.07 | down | Tmem14a | AK017734 |
| A_55_P2044432 | 0.00876 | 2.78 | down | Trav6d-6 | M16676 |
| A_55_P2160686 | 0.02911 | 2.14 | down | Tsc22d1 | NM_207652 |
| A_51_P245503 | 0.00016 | 2.31 | down | Ugt2b1 | NM_152811 |
| A_52_P260555 | 0.00653 | 2.82 | down | Vil1 | NM_009509 |
| A_55_P1973447 | 0.04120 | 2.31 | down | Ybx2 |  |
| A_51_P249302 | 0.04954 | 4.35 | up | Abcd2 | NM_011994 |
| A_55_P2029366 | 0.00599 | 2.59 | up | Abhd6 | NM_025341 |
| A_52_P423174 | 0.00038 | 4.00 | up | Acaa1b | NM_146230 |
| A_55_P2104532 | 0.03258 | 2.32 | up | Acacb | NM_133904 |
| A_55_P2165091 | 0.01872 | 2.42 | up | Acnat2 | NM_145368 |
| A_55_P2038358 | 0.00259 | 8.96 | up | Acot1 | NM_012006 |
| A_52_P525183 | 0.00011 | 31.39 | up | Acot2 | NM_134188 |
| A_55_P2069907 | 0.03152 | 19.02 | up | Acot3 | NM_134246 |
| A_52_P215170 | 0.01836 | 3.17 | up | Acot4 | NM_134247 |
| A_55_P2038362 | 0.00019 | 10.83 | up | Acot5 | NM_145444 |
| A_51_P191354 | 0.00049 | 3.54 | up | Acot6 | AK014558 |
| A_52_P57622 | 0.01356 | 3.56 | up | Acss3 | NM_198636 |
| A_52_P205255 | 0.00212 | 2.53 | up | Adam11 | NM_001110778 |
| A_66_P106760 | 0.00237 | 4.82 | up | Adam32 | NM_153397 |
| A_55_P1968433 | 0.00040 | 2.35 | up | Agpat9 | NM_172715 |
| A_55_P2002854 | 0.00339 | 2.12 | up | Aifm2 | NM_001039194 |
| A_55_P2122633 | 0.04837 | 2.06 | up | Airn | AK032756 |
| A_51_P331288 | 0.00174 | 3.95 | up | Akr1b7 | NM_009731 |
| A_55_P2163098 | 0.00020 | 7.34 | up | Akr1c18 | NM_134066 |
| A_55_P2180899 | 0.00136 | 3.65 | up | Aldh3a2 | NM_007437 |
| A_51_P176972 | 0.02184 | 4.26 | up | Amigo2 | NM_178114 |
| A_51_P338443 | 0.00053 | 2.05 | up | Angptl4 | NM_020581 |
| A_51_P442206 | 0.01298 | 2.02 | up | Ankrd42 | NM_028665 |
| A_55_P2122709 | 0.02111 | 2.19 | up | Arhgef3 | NM_027871 |
| A_55_P2133220 | 0.00002 | 17.06 | up | Arhgef39 | NM_001013377 |
| A_51_P330213 | 0.00018 | 2.93 | up | Asf1b | NM_024184 |
| A_55_P1988228 | 0.00122 | 6.81 | up | Aspm | NM_009791 |
| A_52_P452689 | 0.00488 | 7.38 | up | Atf3 | NM_007498 |
| A_52_P616332 | 0.00215 | 2.02 | up | Atp10d | NM_153389 |
| A_55_P1980636 | 0.00067 | 4.57 | up | Aurka | NM_011497 |
| A_55_P2340448 | 0.00062 | 4.45 | up | B230114P17Rik | AK045415 |
| A_55_P2186648 | 0.00760 | 2.82 | up | Bard1 | NM_007525 |
| A_55_P2171493 | 0.01324 | 2.64 | up | BC030867 | NM_153544 |
| A_52_P340669 | 0.00101 | 7.06 | up | Bhlha15 | NM_010800 |
| A_55_P1983768 | 0.00075 | 7.39 | up | Birc5 | NM_009689 |
| A_55_P1972322 | 0.00387 | 2.14 | up | Btg3 | NM_009770 |
| A_51_P117581 | 0.02410 | 2.03 | up | Cables1 | NM_022021 |
| A_55_P1994020 | 0.00475 | 2.53 | up | Casc4 | NM_001205369 |
| A_55_P1995135 | 0.02954 | 2.51 | up | Casz1 | NM_027195 |
| A_51_P380750 | 0.00537 | 2.31 | up | Cbfa2t3 | NM_009824 |
| A_52_P661587 | 0.00579 | 2.04 | up | Ccdc141 | NM_001025576 |
| A_51_P481920 | 0.00046 | 5.97 | up | Ccna2 | NM_009828 |
| A_55_P2065671 | 0.00483 | 5.14 | up | Ccnb1 | NM_172301 |
| A_51_P457528 | 0.00033 | 4.07 | up | Ccnb2 | NM_007630 |
| A_51_P375146 | 0.00025 | 7.80 | up | Cd36 | NM_007643 |
| A_55_P1996946 | 0.00053 | 12.07 | up | Cdc20 | NM_023223 |
| A_52_P211223 | 0.00195 | 4.50 | up | Cdca2 | NM_175384 |
| A_52_P628067 | 0.00092 | 4.31 | up | Cdca3 | NM_013538 |
| A_51_P125135 | 0.00003 | 4.37 | up | Cdca5 | NM_026410 |
| A_51_P155142 | 0.00051 | 2.31 | up | Cdca8 | NM_026560 |
| A_55_P2048588 | 0.00765 | 3.50 | up | Cdk1 | NM_007659 |
| A_51_P363947 | 0.01086 | 3.99 | up | Cdkn1a | NM_007669 |
| A_52_P30989 | 0.01246 | 4.85 | up | Cdkn3 | NM_028222 |
| A_51_P164014 | 0.00191 | 8.62 | up | Cenpe | NM_173762 |
| A_51_P228171 | 0.00143 | 3.09 | up | Cenpp | NM_025495 |
| A_55_P2041614 | 0.00491 | 6.65 | up | Cerkl | NM_001048176 |
| A_51_P196973 | 0.01581 | 2.58 | up | Chaf1a | NM_013733 |
| A_55_P2177513 | 0.00319 | 2.33 | up | Chrna2 | NM_144803 |
| A_51_P208680 | 0.00401 | 3.72 | up | Chtf18 | NM_145409 |
| A_55_P1969131 | 0.00034 | 3.91 | up | Cidec | NM_178373 |
| A_52_P56397 | 0.00210 | 9.02 | up | Clhc1 | NM_001081099 |
| A_55_P2180744 | 0.00213 | 2.84 | up | Clstn3 | NM_153508 |
| A_51_P300506 | 0.00382 | 2.34 | up | Cox6b2 | NM_183405 |
| A_55_P1956882 | 0.00231 | 5.05 | up | Cpt1b | NM_009948 |
| A_55_P1983314 | 0.00737 | 3.44 | up | Crat | NM_007760 |
| A_66_P123635 | 0.02424 | 2.15 | up | Csf2rb | NM_007780 |
| A_55_P2034663 | 0.01812 | 2.06 | up | Csf2rb2 | NM_007781 |
| A_55_P1997126 | 0.00049 | 13.18 | up | Ctse | NM_007799 |
| A_55_P2044653 | 0.02138 | 5.58 | up | Cyp2b10 | NM_009999 |
| A_52_P366803 | 0.01642 | 5.12 | up | Cyp3a44 | NM_177380 |
| A_55_P2011111 | 0.00005 | 21.93 | up | Cyp4a10 | NM_010011 |
| A_51_P238576 | 0.00007 | 42.66 | up | Cyp4a14 | NM_007822 |
| A_55_P1952399 | 0.00006 | 9.03 | up | Cyp4a31 | NM_001252539 |
| A_55_P1987620 | 0.00015 | 14.88 | up | Cyp4a32 | NM_001100181 |
| A_55_P2092993 | 0.00000 | 7.14 | up | D330045A20Rik | NM_175326 |
| A_52_P269461 | 0.00352 | 2.77 | up | Ddhd2 | NM_028102 |
| A_52_P533146 | 0.00983 | 2.08 | up | Ddit3 | NM_007837 |
| A_51_P245796 | 0.04425 | 2.34 | up | Ddit4 | NM_029083 |
| A_55_P2159189 | 0.00133 | 2.02 | up | Decr1 | NM_026172 |
| A_52_P321140 | 0.00135 | 3.35 | up | Defb1 | NM_007843 |
| A_51_P352381 | 0.00196 | 2.13 | up | Dhrs4 | NM_030686 |
| A_51_P198835 | 0.00134 | 2.51 | up | Dnajb9 | NM_013760 |
| A_55_P1985835 | 0.00882 | 2.33 | up | Dnase1 | NM_010061 |
| A_55_P1992839 | 0.01088 | 2.69 | up | Dsn1 | NM_025853 |
| A_51_P263246 | 0.00075 | 18.14 | up | Dusp8 | NM_008748 |
| A_52_P232580 | 0.00232 | 2.37 | up | Dyrk3 | NM_145508 |
| A_51_P421846 | 0.00155 | 2.18 | up | Ech1 | NM_016772 |
| A_51_P105589 | 0.00003 | 2.24 | up | Eci1 | NM_010023 |
| A_55_P1976127 | 0.02182 | 4.03 | up | Ect2 | NM_007900 |
| A_51_P462918 | 0.00001 | 9.55 | up | Ehhadh | NM_023737 |
| A_51_P505493 | 0.00938 | 2.00 | up | Elovl5 | NM_134255 |
| A_55_P2071811 | 0.02112 | 2.24 | up | Eml2 | NM_028153 |
| A_51_P126437 | 0.00473 | 3.27 | up | Enc1 | NM_007930 |
| A_65_P20641 | 0.00508 | 2.54 | up | Fads2 | AK215784 |
| A_66_P101724 | 0.02152 | 2.86 | up | Fam124a | NM_001243857 |
| A_51_P137184 | 0.00968 | 2.28 | up | Fam209 | NM_029608 |
| A_52_P330214 | 0.02009 | 2.01 | up | Fanca | NM_016925 |
| A_52_P100252 | 0.01683 | 2.19 | up | Fasn | NM_007988 |
| A_55_P2027879 | 0.01394 | 3.08 | up | Fbf1 | NM_172571 |
| A_52_P353541 | 0.01968 | 2.53 | up | Fbxw13 | NM_177598 |
| A_52_P235347 | 0.00145 | 10.39 | up | Fgf21 | NM_020013 |
| A_52_P294510 | 0.02173 | 2.39 | up | Fgl1 | BC029734 |
| A_55_P2110245 | 0.00130 | 7.01 | up | Fignl1 | NM_001163359 |
| A_51_P164630 | 0.01823 | 2.52 | up | Fitm1 | NM_026808 |
| A_55_P2035122 | 0.00111 | 2.31 | up | Fitm2 | AK052945 |
| A_55_P2113051 | 0.04740 | 2.31 | up | Fosb | NM_008036 |
| A_52_P28806 | 0.00358 | 3.39 | up | Foxm1 | NM_008021 |
| A_52_P681391 | 0.04828 | 2.48 | up | G0s2 | NM_008059 |
| A_51_P187082 | 0.01652 | 2.79 | up | G6pdx | NM_008062 |
| A_52_P1197913 | 0.03095 | 2.97 | up | Gadd45b | NM_008655 |
| A_55_P2013823 | 0.00212 | 6.07 | up | Gal3st1 | NM_016922 |
| A_55_P1960735 | 0.00148 | 7.00 | up | Gdf15 | NM_011819 |
| A_55_P2083180 | 0.02009 | 3.23 | up | Gm10272 | NR_026831 |
| A_55_P2102065 | 0.00276 | 4.16 | up | Gm10639 | NM_001122660 |
| A_55_P2011436 | 0.00022 | 2.07 | up | Gm11223 | AK030082 |
| A_55_P2148674 | 0.00384 | 5.54 | up | Gm14762 | XR_106038 |
| A_66_P134984 | 0.00283 | 10.98 | up | Gm15441 | NR_040409 |
| A_55_P2032946 | 0.00098 | 8.27 | up | Gm3776 | NM_001243092 |
| A_55_P2094713 | 0.02789 | 2.29 | up | Gm4736 | NM_053251 |
| A_51_P342481 | 0.00118 | 2.18 | up | Gpd2 | NM_010274 |
| A_52_P141687 | 0.02279 | 2.51 | up | Gpr37l1 | NM_134438 |
| A_55_P2170454 | 0.00518 | 2.20 | up | Gsta2 | NM_008182 |
| A_55_P2102060 | 0.00074 | 2.53 | up | Gstm3 | NM_010359 |
| A_51_P350048 | 0.00020 | 2.24 | up | Gstt2 | NM_010361 |
| A_55_P1997106 | 0.00664 | 2.61 | up | Gyltl1b | NM_172670 |
| A_51_P159612 | 0.00302 | 4.29 | up | Hebp2 | NM_019487 |
| A_51_P270949 | 0.03053 | 2.19 | up | Hist1h1b | NM_020034 |
| A_52_P420466 | 0.02731 | 2.10 | up | Hist1h2ab | NM_175660 |
| A_51_P263965 | 0.03775 | 2.05 | up | Hmox1 | NM_010442 |
| A_52_P132165 | 0.00016 | 2.76 | up | Hsd17b11 | NM_053262 |
| A_55_P2080151 | 0.02752 | 2.39 | up | Hspa2 | NM_008301 |
| A_55_P2039284 | 0.04225 | 2.22 | up | Hspb1 | NM_013560 |
| A_55_P1967539 | 0.01609 | 2.12 | up | Hunk | NM_015755 |
| A_55_P2085776 | 0.00012 | 3.86 | up | Ifi27l2b | NM_145449 |
| A_51_P367060 | 0.00708 | 2.20 | up | Ifrd1 | NM_013562 |
| A_51_P447545 | 0.00468 | 4.54 | up | Igfbp1 | NM_008341 |
| A_55_P1962400 | 0.00607 | 2.28 | up | Il1rn | NM_001039701 |
| A_51_P459108 | 0.02677 | 2.33 | up | Insl6 | NM_013754 |
| A_55_P2158990 | 0.00442 | 5.72 | up | Jun | NM_010591 |
| A_55_P2091116 | 0.01681 | 2.42 | up | Kbtbd12 | NM_001142724 |
| A_51_P107782 | 0.00126 | 2.31 | up | Kdsr | NM_027534 |
| A_51_P133137 | 0.01017 | 6.70 | up | Kif20a | NM_009004 |
| A_55_P2109717 | 0.00154 | 4.01 | up | Kif20b | NM_183046 |
| A_55_P2056654 | 0.00047 | 2.25 | up | Kif22 | NM_145588 |
| A_52_P281659 | 0.02331 | 2.46 | up | Klf13 | AK002926 |
| A_51_P245414 | 0.00975 | 2.44 | up | Klk1 | NM_010639 |
| A_55_P2112986 | 0.02988 | 2.38 | up | Klk1b22 | NM_010114 |
| A_66_P130035 | 0.03293 | 2.03 | up | Klk1b24 | NM_010643 |
| A_55_P2025006 | 0.00976 | 2.22 | up | Klk1b26 | NM_010644 |
| A_51_P413147 | 0.00811 | 2.45 | up | Klk1b3 | NM_008693 |
| A_51_P295034 | 0.00942 | 2.48 | up | Klk1b4 | NM_010915 |
| A_55_P2002933 | 0.00486 | 2.39 | up | Klk1b5 | NM_008456 |
| A_51_P155503 | 0.00628 | 2.55 | up | Klk1b8 | NM_008457 |
| A_55_P2158011 | 0.00003 | 7.26 | up | Knstrn | NM_026412 |
| A_51_P287198 | 0.00297 | 6.52 | up | Krt23 | NM_033373 |
| A_55_P1973930 | 0.00925 | 2.07 | up | Krt79 | NM_146063 |
| A_55_P2070869 | 0.01806 | 11.29 | up | Lcn2 | NM_008491 |
| A_55_P2192662 | 0.00123 | 8.97 | up | Lepr | NM_001122899 |
| A_51_P497100 | 0.00209 | 2.71 | up | Lgals4 | NM_010706 |
| A_52_P154741 | 0.00002 | 4.45 | up | Lgals6 | NM_010707 |
| A_51_P440047 | 0.00825 | 2.02 | up | Lgalsl | NM_173752 |
| A_55_P1953991 | 0.01567 | 2.01 | up | Lhb | NM_008497 |
| A_55_P2094158 | 0.00418 | 2.17 | up | Lhx6 | NM_008500 |
| A_55_P1979639 | 0.02088 | 2.08 | up | LOC101055842 | XM_003946251 |
| A_66_P138976 | 0.01056 | 2.17 | up | Lpin2 | NM_001164885 |
| A_51_P267354 | 0.01286 | 2.17 | up | Lrfn3 | NM_175478 |
| A_52_P11402 | 0.00436 | 2.46 | up | Lrrc24 | NM_198119 |
| A_51_P343517 | 0.00036 | 34.61 | up | Ly6d | NM_010742 |
| A_51_P233160 | 0.02511 | 3.05 | up | Lysmd2 | NM_027309 |
| A_55_P2090060 | 0.00002 | 12.39 | up | Mab21l3 | NM_172295 |
| A_55_P2133255 | 0.00060 | 2.33 | up | Mad2l1 | NM_019499 |
| A_51_P397296 | 0.00524 | 2.99 | up | Marveld3 | NM_028584 |
| A_55_P2130885 | 0.01519 | 2.40 | up | Mas1 | NM_008552 |
| A_51_P190111 | 0.01121 | 3.31 | up | Mcm5 | NM_008566 |
| A_55_P2171413 | 0.00270 | 2.64 | up | Me1 | NM_001198933 |
| A_51_P141104 | 0.04267 | 2.14 | up | Med18 | NM_026039 |
| A_51_P279437 | 0.01825 | 3.96 | up | Mfsd2a | NM_029662 |
| A_51_P215077 | 0.01528 | 2.05 | up | Mgst3 | NM_025569 |
| A_55_P2073377 | 0.00008 | 5.44 | up | Mki67 | NM_001081117 |
| A_55_P2037428 | 0.00001 | 14.57 | up | Mogat1 | NM_026713 |
| A_52_P167278 | 0.01201 | 2.44 | up | Mthfd1l | NM_172308 |
| A_52_P656845 | 0.00984 | 2.14 | up | Mtnr1a | NM_008639 |
| A_51_P302520 | 0.00446 | 5.44 | up | Myom1 | NM_010867 |
| A_51_P501018 | 0.01728 | 2.62 | up | Nek2 | NM_010892 |
| A_55_P2039086 | 0.04685 | 2.81 | up | Nfkbiz | NM_030612 |
| A_55_P2135311 | 0.00509 | 2.05 | up | Nol3 | NM_030152 |
| A_55_P1985433 | 0.01075 | 4.57 | up | Nrg1 | NM_178591 |
| A_55_P2115330 | 0.00455 | 5.16 | up | Nrg4 | AK080089 |
| A_65_P15245 | 0.00341 | 2.25 | up | Nrp2 | NM_001077406 |
| A_51_P296796 | 0.04113 | 2.12 | up | Nt5dc2 | NM_027289 |
| A_51_P240453 | 0.00179 | 6.01 | up | Nusap1 | NM_133851 |
| A_51_P196844 | 0.04415 | 3.18 | up | Osbpl3 | NM_027881 |
| A_55_P2241299 | 0.00634 | 2.25 | up | Otud7b | NM_001025613 |
| A_55_P2028015 | 0.00380 | 2.03 | up | Pawr | NM_054056 |
| A_51_P230098 | 0.00005 | 9.39 | up | Pbk | NM_023209 |
| A_52_P136709 | 0.00172 | 2.59 | up | Pctp | NM_008796 |
| A_65_P12530 | 0.00053 | 7.04 | up | Pde4d | NM_011056 |
| A_51_P350453 | 0.00071 | 7.77 | up | Pdk4 | NM_013743 |
| A_55_P2171378 | 0.00250 | 2.33 | up | Pdzk1ip1 | NM_001164557 |
| A_51_P456957 | 0.00135 | 2.37 | up | Pex11a | NM_011068 |
| A_51_P344566 | 0.00038 | 12.19 | up | Plk1 | NM_011121 |
| A_55_P1993955 | 0.00120 | 2.97 | up | Pltp | NM_011125 |
| A_51_P208145 | 0.02388 | 3.05 | up | Pmel | NM_021882 |
| A_55_P1972720 | 0.00159 | 2.26 | up | Pmm1 | NM_013872 |
| A_55_P2025514 | 0.00369 | 7.38 | up | Pnpla3 | NM_054088 |
| A_51_P155873 | 0.00483 | 7.43 | up | Ppp1r3g | NM_029628 |
| A_55_P2001028 | 0.04181 | 2.01 | up | Ppp2r3c | NM_021529 |
| A_55_P1988083 | 0.00021 | 15.94 | up | Prc1 | NM_145150 |
| A_51_P389531 | 0.03549 | 2.06 | up | Prmt1 | NM_019830 |
| A_51_P209818 | 0.00271 | 3.61 | up | Prtn3 | NM_011178 |
| A_55_P2052290 | 0.01670 | 2.66 | up | Psat1 | NM_001205339 |
| A_55_P2183433 | 0.00306 | 3.54 | up | Rab30 | NM_029494 |
| A_55_P2127702 | 0.00039 | 3.55 | up | Racgap1 | NM_001253809 |
| A_51_P148105 | 0.00098 | 4.57 | up | Rad51 | NM_011234 |
| A_55_P1991688 | 0.00031 | 4.70 | up | Rad51ap1 | NM_009013 |
| A_51_P269792 | 0.00016 | 47.29 | up | Rad51b | NM_009014 |
| A_55_P2091928 | 0.03618 | 3.32 | up | Raet1b | NM_009017 |
| A_55_P2058550 | 0.00384 | 4.30 | up | Raet1c | NM_009018 |
| A_55_P2115955 | 0.03317 | 4.39 | up | Raet1e | NM_198193 |
| A_55_P1961736 | 0.00039 | 2.84 | up | Rcan2 | NM_207649 |
| A_52_P240796 | 0.00014 | 3.36 | up | Rdh16 | NM_009040 |
| A_51_P319070 | 0.00322 | 2.56 | up | Retsat | NM_026159 |
| A_51_P496309 | 0.02911 | 3.02 | up | Rfx4 | NM_001024918 |
| A_51_P249286 | 0.00531 | 6.75 | up | Rgs16 | NM_011267 |
| A_52_P89567 | 0.00989 | 2.79 | up | Rhob | NM_007483 |
| A_51_P331870 | 0.00754 | 2.17 | up | Rnf145 | NM_028862 |
| A_51_P263302 | 0.01375 | 2.96 | up | Rnf24 | NM_178607 |
| A_52_P379337 | 0.00946 | 2.12 | up | Rtn4 | NM_194054 |
| A_55_P2031471 | 0.00017 | 12.33 | up | Rufy4 | NM_001034060 |
| A_55_P2065991 | 0.01349 | 2.21 | up | S100a11 | NM_016740 |
| A_51_P256827 | 0.01751 | 2.28 | up | S100a8 | NM_013650 |
| A_55_P2013236 | 0.00010 | 134.41 | up | S100g | NM_009789 |
| A_51_P472217 | 0.04873 | 4.31 | up | Sapcd2 | NM_001081085 |
| A_52_P682382 | 0.00034 | 3.45 | up | Scd1 | NM_009127 |
| A_55_P2099594 | 0.00036 | 4.27 | up | Scd3 | NM_024450 |
| A_55_P1973844 | 0.00325 | 6.11 | up | Sema5b | NM_013661 |
| A_55_P1994694 | 0.00108 | 2.95 | up | Serinc2 | NM_001253386 |
| A_51_P162955 | 0.00092 | 15.91 | up | Serpina7 | NM_177920 |
| A_51_P487999 | 0.00586 | 7.33 | up | Sgol1 | NM_028232 |
| A_51_P204402 | 0.00136 | 5.08 | up | Shcbp1 | NM_011369 |
| A_55_P2044242 | 0.01239 | 2.43 | up | Slc13a5 | NM_001004148 |
| A_55_P1960828 | 0.01344 | 2.67 | up | Slc16a13 | NM_172371 |
| A_52_P163021 | 0.00206 | 2.08 | up | Slc17a8 | NM_182959 |
| A_51_P407774 | 0.01077 | 2.18 | up | Slc22a21 | NM_019723 |
| A_55_P1991841 | 0.00253 | 4.92 | up | Slc22a27 | NM_134256 |
| A_51_P165060 | 0.00222 | 3.48 | up | Slc22a5 | NM_011396 |
| A_52_P286520 | 0.00000 | 3.20 | up | Slc23a2 | NM_018824 |
| A_51_P344008 | 0.00168 | 2.08 | up | Slc25a20 | NM_020520 |
| A_52_P662244 | 0.00304 | 2.09 | up | Slc25a47 | NM_001012310 |
| A_51_P217498 | 0.03142 | 6.45 | up | Slc2a4 | NM_009204 |
| A_55_P1985351 | 0.00740 | 6.99 | up | Slc35f2 | NM_028060 |
| A_55_P1959828 | 0.00375 | 2.07 | up | Slc35g1 | NM_175507 |
| A_51_P215489 | 0.00390 | 2.85 | up | Slc37a1 | NM_153062 |
| A_51_P206235 | 0.00185 | 5.93 | up | Slc39a5 | NM_028051 |
| A_55_P1989653 | 0.04046 | 3.52 | up | Slco4a1 | NM_148933 |
| A_55_P1965154 | 0.00601 | 2.95 | up | Spc25 | NM_001199123 |
| A_52_P381484 | 0.03387 | 2.03 | up | Spon2 | NM_133903 |
| A_51_P396983 | 0.01319 | 4.54 | up | Ssu2 | NM_175525 |
| A_51_P352594 | 0.00329 | 2.04 | up | St5 | NM_001001326 |
| A_55_P2043862 | 0.00011 | 2.18 | up | Stmn1 | NM_019641 |
| A_52_P293682 | 0.00577 | 2.63 | up | Sult2a7 | NM_001184981 |
| A_51_P273538 | 0.01005 | 2.19 | up | Syce2 | NM_027954 |
| A_55_P2004797 | 0.00015 | 2.71 | up | Tacc2 | NM_001004468 |
| A_55_P2004801 | 0.00012 | 2.34 | up | Tacc3 | NM_001040435 |
| A_55_P2159234 | 0.00585 | 2.59 | up | Tatdn2 | NM_001033463 |
| A_55_P2047163 | 0.04471 | 2.07 | up | Tmc5 | NM_001105252 |
| A_55_P2105359 | 0.01679 | 2.18 | up | Tmed5 | AK140979 |
| A_66_P118772 | 0.01221 | 3.63 | up | Tmem136 | NM_001034863 |
| A_66_P136998 | 0.00117 | 3.71 | up | Tmem43 | NM_028766 |
| A_51_P368496 | 0.00480 | 2.73 | up | Tmem98 | NM_029537 |
| A_51_P288916 | 0.01444 | 2.60 | up | Tmtc2 | NM_177368 |
| A_55_P1981972 | 0.02751 | 3.52 | up | Tnfrsf13b | NM_021349 |
| A_51_P369200 | 0.00045 | 9.27 | up | Tpx2 | NM_028109 |
| A_51_P254045 | 0.03418 | 3.51 | up | Traip | NM_011634 |
| A_55_P2033272 | 0.00073 | 3.30 | up | Treh | NM_021481 |
| A_55_P2069597 | 0.00152 | 2.08 | up | Tspan17 | NM_028841 |
| A_55_P2006255 | 0.00376 | 2.82 | up | Txnip | NM_001009935 |
| A_52_P338066 | 0.00378 | 2.40 | up | Ubd | NM_023137 |
| A_51_P451151 | 0.00370 | 7.36 | up | Ube2c | NM_026785 |
| A_51_P297105 | 0.00025 | 2.97 | up | Ucp2 | NM_011671 |
| A_66_P108247 | 0.00002 | 3.06 | up | Ucp3 | NM_009464 |
| A_55_P2035286 | 0.01664 | 5.04 | up | Uhrf1 | NM_010931 |
| A_55_P1973770 | 0.00061 | 3.10 | up | Unc5b | NM_029770 |
| A_55_P2073313 | 0.00586 | 5.06 | up | Unc79 | NM_001081017 |
| A_55_P2030524 | 0.00181 | 4.49 | up | Vldlr | NM_013703 |
| A_51_P424532 | 0.00058 | 10.08 | up | Vnn1 | NM_011704 |
| A_52_P481316 | 0.00615 | 3.16 | up | Wdr93 | NM_001037927 |
| A_52_P495553 | 0.04687 | 2.81 | up | Zbtb10 | NM_177660 |
| A_51_P464064 | 0.00157 | 2.14 | up | Zc3h13 | NM_026083 |
| A_55_P1964433 | 0.01362 | 2.38 | up | Zmynd12 | NM_001014900 |
| A_52_P59228 | 0.00565 | 2.65 | up | Zswim3 | NM_178375 |
| A_55_P2040743 | 0.00639 | 4.24 | up | Zwilch | NM_026507 |
